# Comparison of Ante-Mortem Clinical Diagnosis and Final Autopsy Diagnosis: Experience from a Single Academic Centre in Pretoria, South Africa

**DOI:** 10.3390/diseases12100229

**Published:** 2024-09-27

**Authors:** Lesedi Makgwethele Nevondo, Tebatso Kekana, Khomotso Comfort Maaga, Moshawa Calvin Khaba

**Affiliations:** 1 Department of Anatomical Pathology, Dr George Mukhari Tertiary Laboratory, National Health Laboratory Service, Sefako Makgatho Health Sciences University, Ga-Rankuwa 0208, South Africa; hlopolosa@yahoo.com (L.M.N.); tebatso.kekana@nhls.ac.za (T.K.); 2 Department of Public Health, Sefako Makgatho Health Sciences University, Ga-Rankuwa 0208, South Africa; khomotso.maaga@smu.ac.za

**Keywords:** autopsy, ante-mortem diagnosis, discrepancies, clinical audit

## Abstract

**Background/Objectives**: There seems to be a global reduction in the number of clinical post-mortems requested and performed worldwide, suggesting a decreasing need for post-mortem examinations. Despite advances in medical technology, autopsies remain a relevant tool to determine cause of death. **Methods**: A total of 276 post-mortem results were extracted from the NHLS lab track database, of which only 152 were included in this study. Discrepancies between ante and post-mortem diagnoses were evaluated using the Goldman classification. Data were analysed using STATA-18. **Results**: The sample consisted largely of females (*n* = 101, 66.45%) aged 30 and above (*n* = 58, 33.80%), with a mean age of 28.3. Of the 152 samples analysed, 60% (*n* = 92) of all postmortems showed a correlation between ante- and post-mortem diagnoses. However, 29.1% (*n* = 45) of cases showed major discrepancies which could have been prevented if correct diagnoses were made. Metabolic diseases were most frequently misdiagnosed (*p* = 0.020), with more cases of Class I discrepancies than Class V discrepancies (15.5% (*n* = 7) vs. 2.1% (*n* = 2), respectively. Additionally, infections (*n* = 59; 39%) were the most common cause of death. **Conclusions**: Even with marked improvements in diagnostic technology, a post-mortem examination is a necessary quality control tool that can be used to verify cause of death, and thus improve clinical practice.

## 1. Introduction

A post-mortem examination, also referred to as an autopsy, is defined as the dissection and examination of a dead body to determine cause of death. Medico-legal/forensic autopsies and clinical autopsies constitute two types of autopsies. Medico-legal autopsies are performed in patients who die from unnatural causes. In contrast, clinical autopsies are performed in patients with unknown or undiagnosed causes of death [1]. Although the improvement in the accuracy of clinical diagnoses through modern technology suggests a decreased need for clinical autopsies, there is evidence to support that this thinking does not align with practice in the absolute. The essential role of post-mortem examinations is highlighted during large epidemics, as they are useful in identifying new disease entities and the re-emergence of known disease entities [2]. Autopsy has also shown multiple benefits in diagnosing new disease entities, and in elucidating disease mechanisms and pathological processes. Notwithstanding, there has been a global decrease in the number of clinical autopsies requested, which may indicate a declining necessity, although this is not entirely accurate [1,3].

Additionally, an autopsy plays an invaluable role as a quality control or clinical audit tool that assists clinicians and health authorities in identifying the most common causes of death in society, thereby devising preventative measures to reduce this particular trend [4,5]. Moreover, an autopsy allows the correct cause of death to be recorded on death certificates, which are further used in research and epidemiological studies, enabling the development of health protocols and policies. This could have significant effects because resources may be committed to managing, preventing, and eradicating incorrect disease entities; thus, this has farther-reaching implications than most would anticipate [4,5].

## 2. Materials and Methods

This was a cross-sectional retrospective study involving autopsies/postmortems performed at our centre from 1 April 2013 to 31 March 2018. 

This study included all clinical autopsies with a clearly defined ante-mortem diagnosis and a final autopsy diagnosis. Medico-legal/forensic autopsies, autopsies with missing clinicopathological data, and autopsies with uncertain causes of death were excluded from this study.

### 2.1. Demographic and Patient Ante-Mortem Diagnoses

Demographic data and favoured ante-mortem diagnoses were retrieved from the National Health Laboratory Service (NHLS) laboratory information system (LIS), TrackCare. Clinicopathological data such as demographics (sex and age), the department requesting the autopsy, the ante-mortem diagnosis, and the autopsy diagnosis were retrieved.

The autopsies were re-assigned with unique numbers. In this study, an adult was defined as any person above the age of 18 years. These data were then captured using Microsoft Excel (Microsoft Office, 2016). To maintain patient confidentiality, a unique number was assigned to each post-mortem sample used in this study. The ante-mortem and autopsy diagnoses were compared and classified according to the Goldman classification. The Goldman classification is a tool used in many studies globally to compare the ante-mortem cause of death with that of the final diagnosis in the post-mortem report. The criteria were divided into Classes I–V. Class I and Class II are considered major discrepancies where the cause of death was missed clinically. In Class I, had the correct diagnosis been made, the patient was likely to have survived with the correct treatment, and in Class II, knowledge of the cause of death would have not altered the outcome. Goldman Classes III and IV are those with minor discrepancies, where the postmortem revealed an incidental finding that may or may not have played a role in the patient outcome. In Goldman Class V, clinical and post-mortem findings are in complete agreement [6].

### 2.2. Selection

In keeping with Goldman classifications I, II, and III, the principal investigator (LMN) and two pathologists (TK, MCK) reviewed all the discrepancies between ante-mortem and autopsy diagnoses.

### 2.3. Data Analysis

Data were analysed using STATA-18 (Stata Corp., College Station, TX, USA). The statistical analysis was performed by constructing frequency tables for the categorical diagnostic variables and calculating the means, medians, and standard deviations for continuous data. Skewness and kurtosis normality tests were used to test for normality of the data, and Pearson’s chi-square test of association was used to explore the factors that were significantly associated with the Goldman classification (*p* < 0.05). Where necessary, continuous data were categorised for the accuracy of chi-square test usage. Finally, all variables were included in the final regression model, whereby ordered logistic regression was performed to control for confounding factors.

## 3. Results

A total of 267 postmortems were requested and performed between 2013 and 2018. Of these, only N = 152 (56.93%) met the inclusion criteria and were analysed further. The normality of the data was tested using the skewness and kurtosis normality tests, and it was found that the data were normally distributed (*p* = 0.01). 

### 3.1. Demographic Profile 

The majority of the participants were aged 30 years and above (*n* = 75, 49.34%), followed by those below the age of 18 (*n* = 51, 33.5%), with a mean age of 28.3. The largest proportion of the sample was female, consisting of 65.64 % (*n* = 101). The top three departments were Paediatrics (*n* = 48, 31.4%), Internal Medicine (*n* = 41, 26.9%), and Obstetrics and Gynaecology (*n* = 40, 26.3%). Further details are provided in Table 1.

### 3.2. Goldman Classification of Cases

**Figure 1:**
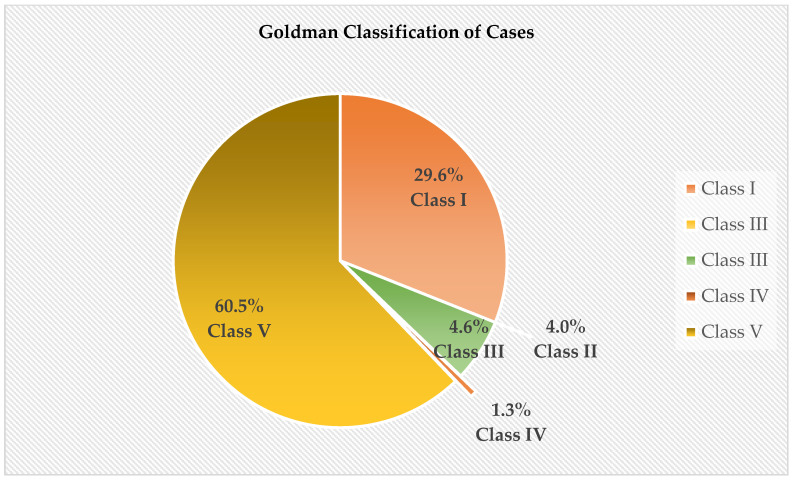
Goldman classification.

Of the 152 cases, there were a total of 51 (33.33%) major discrepancies, consisting of 29.6% (*n* = 45) Class I discrepancies and 4.0% (*n* = 6) Class II discrepancies. Minor discrepancies only consisted of 5.93% (*n* = 9) of cases. Of these, Class III discrepancies, which were classified as missed minor diagnoses related to terminal disease but not related to the cause of death, consisted of 7 (4.61) cases, and Class IV cases made up only 1.3% (*n* = 2) of the cases. A total of 92 (60.5%) cases were classified as Class V, which is in absolute agreement with the Goldman classification. Refer to Figure 1 below:

### 3.3. Clinical Diagnosis

**Figure 2:**
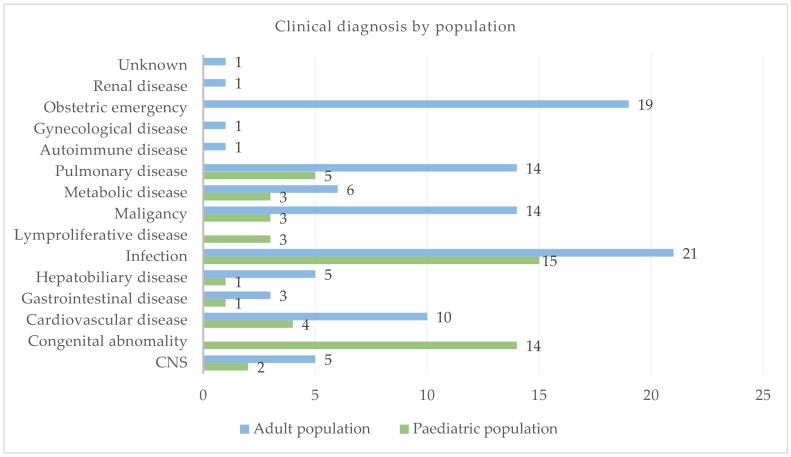
Clinical diagnosis.

Infection (*n* = 15; 29.41%), congenital abnormalities (*n* = 14; 27.45%), and pulmonary diseases (*n* = 5; 9.80%) were the most frequent clinical diagnoses in the paediatric population, whereas infection (*n* = 21; 20.79%), obstetric emergencies (*n* = 19; 18.81%), pulmonary diseases (*n* = 14; 13.86%), and malignancies (*n* = 14; 13.86%) were the most common clinical diagnoses in the adult population (See Figure 2).

### 3.4. Univariate Analysis of Factors Associated with the Goldman Classification

Pearson’s chi-square test of association found that, of the four factors, only two were associated. Age and clinical diagnosis were significantly associated with the Goldman classification. It was found that major discrepancies (Class I) were significantly associated with older age, as those above the age of 30 were more likely to be misdiagnosed (73.3% (*n* = 33) vs. 17.8% (*n* = 8)). In contrast, those below the age of 18 were more likely to be correctly diagnosed, with more cases classified as Class V (45.7% (*n* = 42) vs. 38% (*n* = 35)). Sex and the requesting department had no association. However, clinical diagnosis was also significantly associated with the Goldman classification. Metabolic diseases had higher incidences of Class I discrepancies than Class V (15.5% (*n* = 7) vs. 2.1% (*n* = 2)), thus making these diseases more prone to misdiagnosis. Infections, on the other hand, had fewer incidences of Class I discrepancies than Class V discrepancies (15.5% (*n* = 7) vs. 27.1% (*n* = 25)); therefore, infections were the least likely to be misdiagnosed (See Table 2).

### 3.5. Multivariate Analysis of Factors Associated with the Goldman Classification 

All variables were included in the final ordinal logistic regression model, and only age remained significantly associated, with the paediatric population at 0.5 (50%), with lower odds of Class I discrepancies. Table 3 provides more information below:

### 3.6. Common Causes of Death by Autopsy

**Figure 3:**
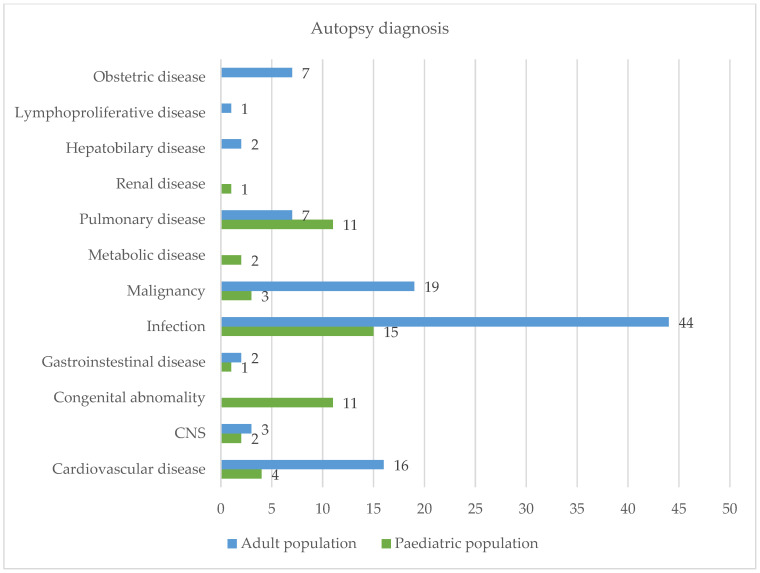
Autopsy diagnosis.

Infection (*n* = 44; 43.56%), malignancy (*n* = 19; 18.81%), and cardiovascular disease (*n* = 16; 15.84%) were the most common causes of death in the adult population, whereas infection (*n* = 15, 30%), congenital abnormalities (*n* = 11; 22%), and pulmonary disease (*n* = 11; 22%) were the most common among the paediatric population. See Figure 3 for further details: 

## 4. Discussion

In South Africa, academic/clinical autopsies performed for natural causes of death are conducted under the Human Tissue Act (Act 65 of 1983), which was incorporated into the National Health Act (61 of 2003), enforced in 2005, and amended in March 2012. If an unnatural death is suspected, the case is reported to the police and further investigations commence [7]. 

According to a survey conducted by Statistics South Africa in 2015, 460,236 deaths occurred when the cause of death was uncertain. In this survey, it was found that post-mortem examination was the most commonly used method to ascertain cause of death [8].

Not many studies have been performed in Africa regarding the trends and discrepancies between ante-mortem and autopsy diagnoses. A study by Mozambique’s Ordi et al. (2019), evaluating the major diagnostic discrepancies in maternal deaths using autopsy, highlighted the indispensable use of the modality as the gold standard for determining cause of death [5]. Thus, a post-mortem examination of a patient can help confirm, clarify, or even correct the ante-mortem diagnosis of the cause of death. Previously, the rate at which postmortems were performed at an institute was used to measure how committed the institute was to providing quality medical care to their community.

Autopsies have also played a significant role in the identification of new diseases, risk factors, and potential disease outbreaks. Although it is assumed that with the improvements in medical technology, including laboratory investigations, cause of death is much easier to ascertain ante mortem, the rates of misdiagnosis and incorrect diagnosis remain the same [9].

In the current study, 267 postmortems were performed over 5 years from April 2013 to March 2018; however, only 152 satisfied the inclusion criteria. This study found that 92 (60.53%) of the 152 cases were correctly diagnosed. This figure is somewhat similar to the 58% reported in a Brazilian study by Rodrigues et al. (2021) [10], but much higher than the 34.4% reported by Joubert and co-authors (2022) [11] in a South African study. The present study further found 51 (33.1%) major discrepancies, consisting of 29.6% (*n* = 45) Class I discrepancies and 4% (*n* = 6) Class II discrepancies, according to the Goldman classification [6]. These high discrepancies are concerning because lower rates have been reported in European countries, with reports ranging from 2 to 15% [12,13,14]. The implication of these Class I discrepancies is that had the correct diagnosis been made and the correct interventions been implemented, there would most likely have been a different outcome regarding patient survival. However, it is important to note that the current study did not consider how much time the patient may have been under medical care and how much time was available for clinical investigations to be performed peri mortem. Only 10 (6.4%) patients showed incidental findings that played a minor or no role in patient outcomes.

The current study observed that more postmortems were performed on adult female patients, of which 48 (31.7%) were requested by Paediatrics, 41 (26.9%) by Internal Medicine, and 40 (26.3%) by Obstetrics and Gynaecology (O&G). The high number of requests in O&G can be explained by the “Saving Mothers” initiative enacted in South Africa, in which a postmortem has to be performed in every case of maternal death [15].

Maternal deaths have been scrutinised in South Africa, especially in terms of the identification of preventable causes of death according to the “Saving the Mothers Health Program”; hence, it is mandatory to perform an autopsy for all maternal deaths [16].

The reason for the disproportionate presentation of the female-to-male ratio in post-mortem cases could also be attributed to poor health-seeking behaviour seen in males. One study attributed this to males’ attitudes in terms of their definition of masculinity that states that men are strong and that “men don’t get sick”. Some men felt that the time wasted in a healthcare facility could have been better used to earn a living, while others were reluctant to discuss their health issues with female medical personnel [17]. The reasons above may explain why there was a higher number of females than males, as two other studies reported contrary findings with a higher male dominance [11,18].

The number of Internal Medicine cases is also not completely unexpected, with South Africa ranking second in the number of deaths attributed to human immunodeficiency virus and acquired immunodeficiency syndrome (HIV/AIDS) worldwide, according to a study performed in 2017 [19]. Unfortunately, data on HIV testing and findings were not available in some of the cases in this study; thus, correlation with HIV status was not considered in this study.

During bivariate analysis, it was found that older age was significantly associated, as those who were 30 years or older were at heightened risk of a Class I misdiagnosis, compared to those 18 years or younger and who were more likely to be accurately diagnosed. The current study’s findings agree with other literature sources [20], as older patients are at risk of a higher incidence of comorbidities that may make it difficult to accurately diagnose due to similar symptoms being present. Sex, however, was not associated, which has also been reported by other authors [20,21,22]. Although it was also found that Internal Medicine had more cases of major misdiagnosis (Class I) than any other department, and Paediatrics had more cases of absolute agreement (Class V), this result was insignificant, showing no association between the classification and the requesting department.

In this study, 59 deaths were related to infections, indicating the need for peri-mortem infection screening test protocols that include microbiological culture and sensitivity. Culture tests should be based on common infections cultured in a given community to reduce the costs of running large panels. The high level of missed infectious diseases may be associated with the lower socioeconomic status of most patients at our referral hospital. These data retrieved from postmortems are of great value, as preventative measures can be undertaken by creating awareness of symptoms and signs through community health education. In addition, through post-mortem data, common pathogens can be identified and tested routinely in cases of suspected infection. This can lead to rapid diagnosis, better patient outcomes, and reduced morbidity and mortality. A high number of HIV infections may also play a role; however, in this study, data on the HIV status of the patients were not available, and further studies in this regard may be considered in the future. Although culture and microbiology are not always routinely performed during post-mortem examinations, there seems to be a need to create protocols for performing cultures in all suspected cases of infections and sepsis as part of routine and common practice. This could lead to policies for testing all critically ill patients upon arrival which will help junior doctors not to miss entities with which they may be unfamiliar. This could even lead to outreach campaigns to educate the public about common signs and symptoms of infectious diseases and the importance of preventive measures, such as washing hands.

After infectious causes, malignancy is the next most common cause of death in adults. Some cases were incorrectly diagnosed as infectious or as other haematological conditions. While malignant tumours were missed in these cases, it is important to note that the patients may have presented with advanced stages of disease and demise before further investigations could be performed, thereby not being able to receive the necessary treatment. Nevertheless, this could lead to deficiencies in the public health system. 

This is a common occurrence in developing countries where patients only seek help at late stages of disease for various reasons, including the availability and affordability of the healthcare system. In South Africa, more than 80% of the population relies on the public health system, which may have dire consequences, especially with the allocation of budgets and resources. Some health centres do not have healthcare workers or medical equipment. The reason for this late presentation may be multifactorial, rather than solely due to misdiagnosis by the attending medical practitioner. 

Some patients may be reluctant to seek medical attention earlier because of long queues and lack of resources [23,24]. Additionally, patients may be seen several times at primary health centres, which may delay proper investigation testing and referral to higher levels of the health system. This may point to the need for improved resources and personnel training at the primary and secondary healthcare levels to improve patient referral time. Continuous training and outreach are necessary to keep medical staff up to date and improve medical officials’ skills in identifying clinical features that prompt the early referral of cases [23,24,25].

Autopsy assessment is also useful in cancer cases, as it could provide family members with closure and arm them with knowledge that could lead to awareness and the early detection of other family members at risk. It could prompt genetic testing in relevant groups and allow early disease detection through frequent screening programs or even prophylactic management, such as bilateral mastectomies or oophorectomies in high-risk patients. 

In the paediatric population, the most misdiagnosed cause of death was attributed to congenital abnormalities, indicating that prenatal screening is an area of concern which requires urgent attention. Most paediatric patients die soon after birth because of complications associated with congenital anomalies. According to maternal healthcare guidelines, level 1 patients (primary healthcare) should have at least one ultrasound performed between 18 and 20 weeks of gestation; however, due to resource constraints, this may not be performed [26]. However costly, policies to make such services available may need to be reconsidered in the future. It is costly for the state to provide medical care, physiotherapy, and other facilities for children with special needs. Providing at least a single prenatal scan for all mothers, not just mothers deemed to be at high risk, allows medical teams to better prepare for the delivery of infants with congenital abnormalities. In addition, with adequate prenatal screening, mothers can make informed decisions about current and future pregnancies. Prenatal screening would also allow families the time to prepare themselves emotionally and financially for any difficulties that they may encounter during pregnancy and postpartum. Additionally, performing post-mortem examinations on infants who die shortly after birth helps give closure to mothers and families who have lost a baby and helps to give healthcare workers foresight on how better to handle the next pregnancy and even possibly how to prevent recurrences of early neonatal death.

Although autopsies are essential for determining cause of death, most causes of misdiagnosis cannot be pinpointed. The causes for misdiagnosis may be multifactorial, consisting of issues with the healthcare system such as limited resources, inadequate referral systems, and competency of medical practitioners, among others. The retrospective nature of this study prevented an explicit examination of this crucial aspect. In light of this, a more comprehensive analysis is required to resolve this matter.

## 5. Conclusions

This study showed that a post-mortem examination is a necessary tool for the accurate diagnosis of cause of death. Although medical technology has improved vastly over the years such that given enough time, most patients can be diagnosed and treated, this study found that in 33.56% of cases, there is a major discrepancy between ante-mortem clinical diagnosis and post-mortem diagnosis. In these cases, loss of life may have been prevented if the correct diagnosis and treatment had been implemented. With this information, doctors working in this district can have a high level of suspicion for infectious and malignant disease in adult patients, and protocols for patient management can be adjusted to prevent such discrepancies in the future.

In terms of paediatric deaths, the data collected here suggest that increased sonographic screening should be considered to anticipate and, perhaps, better manage or even prevent deaths caused by congenital anomalies.

From this study and several other studies conducted worldwide, it is clear that there is still room for further improvement in diagnostic medicine to stop unnecessary loss of life. The only way to accomplish this is to understand where the problem lies and the areas in which we are failing to make correct diagnoses. A post-mortem examination seems to be the only feasible method to assess this. Thus, in conclusion, we need to preserve autopsy teaching and continue to develop and improve autopsy skills as an important part of patient care and diagnostic medicine.

## Figures and Tables

**Table 1:** Demographic profile.

Variable	No. of Cases	Percentage (%)
Age (n = 152) (Mean 28.3; SD 22.5; Min 0, Max 85)		
<18	51	33.5
18–29	26	17.7
≥30	75	49.3
Gender (n = 152)		
Female	101	66.5
Male	51	35.6
Requesting Department (n = 152)		
Paediatrics	48	31.5
Internal Medicine	41	26.9
Obstetrics and Gynaecology	40	26.3
General Surgery	9	5.9
Neurosurgery	4	2.6
Emergency	4	2.6
Intensive Care Unit	2	1.3
Cardiothoracic Surgery	2	1.3
Clinical Haematology	1	0.6
Orthopaedics	1	0.6

**Table 2:** Factors associated with the Goldman classification.

Variable	Cases (%)	Goldman Classification					Chi^2^	*p*-Value
		Class I	Class II	Class III	Class IV	Class V		
Age							23.369	0.003 *
<18	51(33.5)	8(17.8)	1(16.7)	3(42.9)	0(0.0)	42(45.7)		
18–29	26(17.7)	4(8.9)	0(0.0)	3(42.9)	0(0.0)	15(16.3)		
≥30	75(49.3)	33(73.3)	5(83.3)	1(14.3)	2(0.0)	35(38.0)		
Gender							2.796	0.592
Female	101(66.5)	32(71.1)	3(50.0)	6(85.7)	1(50.0)	59(63.1)		
Male	51(35.6)	13(28.8)	3(50.0)	1(14.3)	1(50.0)	33(35.9)		
Department							31.310	0.691
Paediatrics	48(31.5)	8(17.8)	1(16.7)	3(42.8)	0(0.0)	36(39.5)		
Internal Medicine	41(26.9)	17(37.8)	1(16.7)	1(14.2)	1(100)	21(23.9)		
Obstetrics and Gynaecology	40(26.3)	13(28.9)	2(33.3)	2(28.6)	1(50.0)	22(24.2)		
General Surgery	9(5.9)	4(8.9)	2(33.3)	0(0.0)	0(0.00)	3(3.3)		
Neurosurgery	4(2.6)	0(0.0)	0(0.0)	0(0.0)	0(0.0)	4(4.40)		
Emergency	4(2.6)	2(4.4)	0(0.0)	1(14.3)	0(0.0)	1(1.0)		
Intensive Care Unit	2(1.3)	1(2.2)	0(0.0)	0(0.0)	0(0.0)	1(1.0)		
Cardiothoracic Surgery	2(1.3)	0(0.0)	0(0.0)	0(0.0)	0(0.0)	2(2.2)		
Clinical Haematology	1(0.6)	0(0.0)	0(0.0)	0(0.0)	0(0.0)	1(1.10)		
Orthopaedics	1(0.6)	0(0.0)	0(0.0)	0(0.0)	0(0.0)	1(1.10)		
Clinical diagnosis							79.919	0.020 *
Autoimmune disease	1(0.6)	1(2.2)	0(0.0)	0(0.0)	0(0.0)	0(0.0)		
CNS	7(4.6)	3(6.6)	0(0.0)	0(0.0)	0(0.0)	4(4.3)		
Cardiovascular disease	14(9.2)	5(11.1)	0(0.0)	0(0.0)	0(0.0)	9(9.7)		
Congenital abnormality	14(9.2)	1(2.2)	0(0.0)	0(0.0)	0(0.0)	13(14.1)		
Gastrointestinal disease	4(2.6)	2(4.4)	0(0.0)	0(0.0)	0(0.0)	2(2.1)		
Gynaecological disease	1(0.7)	0(0.0)	1(16.6)	0(0.0)	0(0.0)	0(0.0)		
Hepatobiliary disease	6(3.9)	2(4.4)	1(16.6)	0(0.0)	0(0.0)	3(3.2)		
Infection	36(23.7)	7(15.5)	1(16.6)	2(28.5)	1(50.0)	25(27.1)		
Lymphoproliferative disease	3(1.9)	1(2.2)	1(16.6)	1(14.2)	0(0.0)	0(0.0)		
Malignancy	17(11.2)	3(6.6)	1(16.6)	1(14.2)	0(0.0)	12(13.0)		
Metabolic disease	9(5.9)	7(15.5)	0(0.0)	0(0.0)	0(0.0)	2(2.1)		
Obstetric emergency	19(12.5)	3(6.6)	0(0.0)	2(28.5)	1(50.0)	13(14.1)		
Pulmonary disease	19(12.5)	9(20.0)	1(16.6)	1(14.2)	0(0.0)	8(8.7)		
Renal disease	1(0.6)	0(0.0)	0(0.0)	0(0.0)	0(0.0)	1(1.0)		
Unknown	1(0.6)	1(2.2)	0(0.0)	0(0.0)	0(0.0)	0(0.0)		

95% CI = confidence interval; *p* < 0.05; * = significantly associated.

**Table 3:** Multivariate analysis of factors associated with the Goldman classification.

Variable	Odds Ratio	Std. Err	*p*-Value	95% Conf. Interval	
Age	0.5356452	0.1264699	0.008 *	0.3372113	0.8508486
Gender	1.04847	0.4424214	0.911	0.4585381	2.397377
Department	1.071671	0.0635736	0.243	0.9540399	1.203806
Clinical diagnosis	1.011896	0.0716671	0.867	0.8807443	1.162577

95% CI = Confidence interval; *p* < 0.05; * = significantly associated.

## Data Availability

All relevant data are included in the article. The original data cannot be shared because of privacy concerns.
